# Attitudes and behaviors regarding online pharmacies in the aftermath of COVID-19 pandemic: At the tipping point towards the new normal

**DOI:** 10.3389/fphar.2022.1070473

**Published:** 2022-12-23

**Authors:** András Fittler, Tünde Ambrus, Anna Serefko, Lenka Smejkalová, Anna Kijewska, Aleksandra Szopa, Mátyás Káplár

**Affiliations:** ^1^ Department of Pharmaceutics, Faculty of Pharmacy, University of Pécs, Pécs, Hungary; ^2^ Department of Applied Pharmacy, Faculty of Pharmacy, Masaryk University, Brno, Czechia; ^3^ Department of Clinical Pharmacy and Pharmaceutical Care, Faculty of Pharmacy, Medical University of Lublin, Lublin, Poland; ^4^ Department of Applied Psychology, Faculty of Humanities and Social Sciences, University of Pécs, Pécs, Hungary

**Keywords:** internet pharmacies, central Europe, online medicine shopping, consumer attitudes and behavior, post-COVID, Visegrad countries (V4), e-pharmacies

## Abstract

The COVID-19 pandemic accelerated the online purchase of goods and services in which today’s e-pharmacy is now an integral part of healthcare provisions in developed countries. The aim of our research was to assess the frequency and attitudes of European consumers purchasing medications online prior to and following the pandemic in the Visegrad Group countries (V4). An online cross-sectional study was conducted between May-August 2022. A 25-item questionnaire with single choice and 5-point Likert scale answers was used and implemented in Google forms and Pollfish. A sampling of 2087 responses was collected. A majority (92.8%) of the respondents were aware medicines can be purchased online, yet prefer traditional pharmacies (4.6 ± 0.8) and somewhat oppose the internet channel (2.8 ± 1.3). Following the outbreak of COVID-19 pandemic, respondents’ attitude towards purchasing consumer goods (3.65 ± 0.89) and medications (3.26 ± 0.91) online increased, however, the change regarding medications is significantly smaller (*p* < 0.01). A distinct increase in the frequency of buying medicines (from 49.16% to 55.48%) and health products (from 60.61% to 63.0%) online was measured since the breakout of the COVID-19 pandemic (*p* < 0.05). Despite the relatively high prevalence witnessed in previous purchases, our results highlight the trend in which 18.3% of participants will definitely purchase medications online while a proportionate 17.7% will not in the future. Although long-term post-COVID attitudes towards the internet pharmacy channel have modestly increased, in comparison to previously published research the frequency of purchasing medications online has significantly increased during the past years. As national legislation in V4 countries permit only non-prescription online sales, our findings are primarily applicable to countries with similar regulatory environment. The rapid global spread of the coronavirus has transitionally affected medicinal purchase behaviors, augmenting potential public health concerns related to online sourcing. Consequently, public awareness campaigns are needed to promote verified online pharmacies and prevent the utilization of illicit websites and the use of unregistered, substandard and falsified medicinal products.

## Introduction

Internet use is a part of everyday life, in which 80% of individuals in the European Union (EU) accessed the internet daily and two out of three Europeans (66%) ordered goods or services over the internet for private use as recorded in 2021 (Eurostat, 2020). Online pharmacies have been a developing channel of the pharmaceutical supply since the beginning of the century. During the early development phase, due to the lack of national regulations and verification systems, low consumer experience, consumer trust, and a relatively low number of legitimate websites, the internet pharmacy market was considered a dubious channel of illegitimate sellers and a source for potentially counterfeit medications. Today, online medicine purchase from legitimate and verified internet pharmacy websites has become an accepted practice among developed countries, especially experienced in major pharmaceutical markets in the United States, Germany and the United Kingdom (Brown and Li, 2014; Cherecheș and Popa, 2021; Lobuteva et al., 2022). As of 2020, the global e-pharmacy market was expected to be valued at 50 billion USD with a compound annual growth rate (CAGAR) of 17% (IQVIA Consumer Health, 2020).

The pandemic has significantly impacted the pharmaceutical market. According to a cross-sectional time series analysis regarding drug purchase data from 68 countries, the number of pharmaceutical units purchased worldwide in March 2020 increased by 15% compared to the previous year, however, following the increase, a rapid correction was observed (Suda et al., 2022). Evidently, the e-pharmacy market underwent increased acceleration due to the pandemic and it has made pharmacy e-commerce integral to healthcare provisions (International Pharmaceutical Federation, 2021), as commerce and healthcare have shifted over to the digital environment. For example, in 2020, the number of products distributed by distance selling pharmacies increased by nearly 45% in the United Kingdom, at the same time, online sales account for a smaller proportion of pharmacy turnover (4.1%) (Wickware, 2021). In numerous countries where online purchasing and home delivery of pharmaceuticals and/or prescription only medications were previously prohibited, governments and authorities have issued specific exemptions for such practices to facilitate patient access to their medications during social distancing, quarantine and isolation (Hammour et al., 2022).

Due to the COVID-19 outbreak caused by SARS-CoV-2, the world has experienced lockdowns and travel restrictions. Regulations and fear of infection have led to an increase in the purchase of services and consumer goods over the internet. As a result of the pandemic, communication between healthcare providers and patients became significantly limited, with most individuals relying on family, friends and the internet for health information, or they managed their health problems on their own. The “typical” motivations (convenience, price comparison and selection) remained decisive, and, due to the pandemic, the main criteria for online shopping among most consumers during the epidemic were price, availability, comfort and hygiene (Gu et al., 2021). The quarantine, social distancing and isolation all related to the COVID-19 pandemic were additional determining factors, however, their relevance diminished in 2022. The digitization which pervades everyday life has also changed shopping habits in the fields of education, employment and social relations, and in parallel with this premise, the “online experience” has increased in connection with expanded products and services.

Online shopping habits, attitudes and frequency have all underwent vast changes, however, due to the special nature of medicines, these changes are somewhat different compared to other products. We assume the frequency of online medicine and health product purchases increased due the pandemic, yet at the same time, this increase remains below the growth rate of e-commerce.

Generally speaking, the Visegrad Group countries are navigating a similar path regarding the regulatory environment of internet pharmacies and distant sale of pharmaceuticals. Following the amendment of national laws aiming at adjusting local legislation to the legislation of the European Union, in terms of pharmaceutical issues, aligned with the judgement handed down by the European Court of Justice in 2003 (Seeberg-Elverfeldt, 2009), all Visegrad Group countries permitted mail-order trade in non-prescription medicines during the first decade of the century. During recent years, all four countries have implemented e-prescription systems, however, the prevalence in use and the maturity of the system varies. E-commerce maturity significantly differs in the observed countries according to a recent market analysis by IQVIA (IQVIA Consumer Health, 2021). The e-commerce market share value from total non-prescription market value has increased from 2019 to 2020 according to the following patterns: from 0.9% to 1.6% in Hungary, from 3.4% to 5.6% in Slovakia, from 4.2% to 5.6% in Poland, and from 8.4% to 12.2% in the Czech Republic (IQVIA Consumer Health, 2021). Information regarding registered pharmacies legally providing mail-order service for medicinal products are available on the websites of national pharmaceutical authorities (see Table 1), and these registered retailers must feature the common logo of the European Commission on their websites (European Medicines Agency, 2022). Mail-order dispensing of non-prescription medications from another country can only be provided by an authorized provider from another EU member state and can only be used for medicines registered in the target country or in the country from which the medicine is ordered. Furthermore, products must be labelled in the official language of the targeted country.

**TABLE 1:** Community and internet pharmacy provision among the Visegrad Group countries.

Country	Population (million)	Brick and mortar community pharmacies^a^		Pharmacies providing distant sale	
		n	Inhabitants per pharmacy	n	Inhabitants per distant selling pharmacy
Czech Republic	10.5	2,683	3,914	158	66,456
Hungary	9.7	2,277	4,267	656	14,786
Poland	37.6	13,000	2,892	168	223,809
Slovakia	5.5	1,856	2,963	89	61,798

^a^ Total number of community pharmacies in the country providing service for outpatients.

Data available from national competent authorities for licensing and administration for pharmaceutical products and pharmaceutical supply.

In European countries, the possibility and structure of non-pharmacy trade (Oleszkiewicz et al., 2021) and distance selling (Seeberg-Elverfeldt, 2009) is determined by individual legal regulations. In the Czech Republic, since 2006, the mail-order dispensing of non-prescription medicines is only provided by a pharmacy with a valid licence. Mail-order dispensing of prescription medicines, compounded medicinal products, and medicines containing narcotics or precursors is forbidden. A pharmacy providing mail-order dispensing is obligated to ensure information services are provided by a pharmacist or a pharmacy technician (Kolář, 2014). Since 2004, the Hungarian national authority has supervised medicine sales and has permitted the operation of online pharmacies. Only non-prescription products can be ordered online by individuals aged 14 years and above, and websites must be operated by an authorized pharmacy including a physical location. As of January 2022, the amendment of the national act on the distribution of medicinal products has prohibited the parcel delivery of over the counter (OTC) medications by couriers (Hungarian Parliament, 2006). Accordingly, only the professional staff of the pharmacy operating the online platform are allowed to deliver pharmaceuticals to patients’ homes, while non-medicinal (health) products such as dietary supplements can be delivered using third party courier services. In Poland, since 2007, it has been possible to mail order medicinal products which do not require a medical prescription from community pharmacies (Polish Government, 2001). Thus, online pharmacies can operate on the condition they simultaneously operate as a stationary business entity. Medicinal products which require a medical prescription are thoroughly excluded from this distribution channel. It is possible to order a prescription drug online, however, such a medicinal product must be collected at the pharmacy, in which the pharmacist will dispense the drug only after receiving the prescription. In Slovakia, the mail-order dispensing of medicines has been possible by authorized community pharmacies since the legislative change was implemented in 2009 (Act No. 402/2009 Coll. changing and supplementing, 2009). Medicines and medical devices dispensed by mail-order pharmacies must be authorized by the law of Slovakia and restricted to OTC medicine. Mail-order pharmacies and medical device stores can also dispense online self-testing diagnostic devices of Class B and C and medical devices of Class I and IIa.

Due to the “self-medication culture” which has been strengthening for decades including an expanding number of illegal traders in the internet drug distribution space, many health risks must be considered (e.g., unreliable health claims and misinformation, therapeutic recommendations which go against evidence-based medical guidelines, side effects, disease progression and prevalent highly controlled substance abuse). The resulting “digital iatrogenesis” is considered a significant public health risk (Mackey and Nayyar, 2016), against which, we can effectively act upon once we understand the motivations and experiences of the population regarding self-medication and the behavior behind online medicine purchasing. Several studies have revealed a link between using online pharmacies and the following sociodemographic factors: a higher level of education, advanced age, higher family income, greater number of prescriptions and/or marital status (Atkinson et al., 2009; Brown and Li, 2014; Fittler et al., 2018; Jairoun et al., 2021). During the past 2 years, only a relatively small number of national (Bowman et al., 2020; Cherecheș and Popa, 2021; Jairoun et al., 2021; Sun et al., 2021; Soboleva et al., 2022) surveys have been conducted, thus, there is little data measuring the effects of the coronavirus epidemic on the prevalence of online purchases and the attitude changes towards internet pharmacies. Evidently, COVID-19 has accelerated trends which were already growing; however, it is debatable if the changes in internet pharmacy utilization will outlast the pandemic.

Our aim is to examine the frequency of online medicine purchases and how it has changed following the coronavirus pandemic. Furthermore, we intend to evaluate consumers’ motivation and attitudes within the framework of an international survey. The focus of our research highlights the Visegrad Group countries in central Europe (Hungary, Czech Republic, Poland and Slovakia), which have a similar legal background in terms of online drug distribution. Our results serve as a useful temporal and geographical benchmark, and the data regarding the population’s internet drug purchasing habits and opinions will contribute to the planning of public health programs by pharmaceutical professional organizations, consumer protection authorities and legislators supporting the conscientious use of medicinal products.

## Materials and methods

### Recruitment and data collection

An online descriptive cross-sectional study was conducted in four central European countries (Czech Republic, Hungary, Poland and Slovakia) representing 12.5% of EU-28, in which the aim was to measure the prevalence and attitudes towards purchasing medications and health products online. Furthermore, to evaluate the change of such purchases prior to and following the outbreak of the coronavirus pandemic in March 2020. These countries have similar indicators regarding socio-demographic characteristics, economics, and cultural backgrounds. Respondents were approached between May and August 2022, *via* the most popular social media platforms throughout the region (Facebook and Instagram) in which the questionnaire was formatted into Google forms. Additionally, the responses were also surveyed through the use of the Pollfish survey research platform. Google forms is a web-based survey administration software commonly used for data collection for self-administered surveys (Google forms, 2022). Pollfish is an internationally accepted research platform using Random Device Engagement (RDE) polling to engage random individuals in their daily activities through any device, in which survey respondents are asked to participate in a poll in exchange for an incentive token (Pollfish, 2022). Reliability of prevalence values calculated in our study and the statistical validity of our survey were achieved by determining a minimum sampling size of 500 responses in each country. Considering the population of the Visegrad Group countries (total 64 million; Czech Republic, Hungary, Poland, and Slovakia with 10.7, 9.7, 38, and 5.5 million inhabitants, respectively), > 500 responses guarantee representative results reflecting the views of the overall population in each country with a 95% confidence level and below 5% margin of error. Study eligibility criteria regarding the participants was 18 years of age or above.

### The survey

Our current questionnaire tool is an updated version of the questionnaire used in our former survey published in 2018 (Fittler et al., 2018), amended with questions regarding online medicine and health product purchases prior to and following the outbreak of the pandemic. The original English language questionnaire was developed by two authors (AF, KM). To avoid bias, forward translations were made to target languages by authors TA, LS, AS, and AF into their mother tongue, while to ensure accuracy, the initial translations were back-translated by independent translators to reveal misunderstandings or unclear wording. In the pilot phase of the survey, we examined the comprehensibility and face validity of the questions with two academic staff from each participating university and a smaller number (*n* = 10) of pilot respondents per country in May 2022. Based on feedback, only minor corrections were necessary prior to live implementation.

The 25-item questionnaire included single choice and 5-point Likert scale answers. It consisted of an introductory paragraph regarding the objectives of the survey, information referencing confidentiality and anonymity followed by four main sections: *Questions related to drug procurement* (*n* = 3); *Attitudes towards online drug purchases* (*n* = 4); *Internet usage habits including medicines and health product purchases* (*n* = 13); *Sociodemographic data* (*n* = 5). The first section is preceded by four screening questions to increase consciousness regarding the attributes of medicines for participants to be able to differentiate medicines from health products (e.g., dietary supplements or herbals) in the later sections of the survey. Participation was voluntarily and anonymous. We did not directly collect data regarding health status, however, respondents were asked if they were taking medications for chronic conditions, and they were offered an opportunity to identify products purchased online including the website they accessed for the purchase. For a detailed list of questions, see the English questionnaire *via* the link in the Data availability statement. National translated versions are available upon request from the Authors.

### Statistical analysis

The surveys were anonymously evaluated. Data were analyzed using the IBM SPSS Statistics 26 program. Descriptive statistics was used to describe respondent characteristics. To analyze the survey data in which ordinal answers were examined, Kurskal-Wallis H and Wilcoxon Z tests were used to analyze scale data ANOVA. The nominal and frequency data were analyzed using Chi-Square analysis in which *p*-values < 0.05 were regarded as statistically significant.

Our study protocol and survey instrument were reviewed and confirmed by the institutional review board (9324 – PTE 2022) at the University of Pecs.

## Results

### Respondent characteristics and internet use

A representative sample of 2087 respondents participated in our study. The number of participants and the margin of error at 95% confidence level for the observed countries include the following: 531 in Czech Republic (4.3%), 504 in Hungary (4.4%), 524 in Poland (4.3%), and 528 in Slovakia (4.3%). A sum of 957 responses was collected *via* Google forms and 1130 *via* Pollfish, see respondent characteristics in Table 2.

**TABLE 2:** Sociodemographic characteristics regarding the international survey respondents (*n* = 2087).

Variable	Group	Frequency				
		Czech Republic *n* = 531	Hungary *n* = 504	Poland *n* = 524	Slovakia *n* = 528	Total *n* = 2115
Sex	Female (%)	260 (49%)	299 (59.3%)	320 (61.1%)	276 (52.3%)	1115 (55.3%)
	Male (%)	266 (50.1%)	199 (39.5%)	202 (38.5)	252 (47.1%)	919 (44.0%)
	Undisclosed	5 (0.9%)	6 (1.2%)	2 (0.4%)	0 (0%)	13 (0.6%)
Age (years)	mean (±SD)	36.1 (13.0)	47.2 (17.5)	37.1 (13.1)	33.2 (12.5)	38.3 (15.1)
	range	18–75	18–88	18–86	18–74	18–88
Education level	Primary school	16 (3.0%)	17 (3.4%)	16 (3.1%)	29 (5.5%)	78 (3.7%)
	Highschool	301 (56.7%)	219 (43.5%)	219 (41.8%)	324 (61.4%)	1063 (50.9%)
	College or University	201 (37.9%)	251 (49.8%)	263 (50.2%)	156 (29.5%)	871 (41.7%)
	PhD/DLA	13 (2.4%)	17 (3.4%)	26 (5.0%)	19 (3.6%)	75 (3.6%)
Residence (inhabitants)	Large city, capitol (>200,000)	132 (24.9%)	149 (29.6%)	175 (33.4%)	64 (12.1%)	520 (24.9%)
	City (50,000–200,000)	142 (26.7%)	151 (30.0%)	158 (30.2%)	112 (21.2%)	563 (27.0%)
	Town (5,000–50,000)	156 (29.4%)	123 (24.4%)	114 (21.8%)	172 (32.6%)	565 (27.1%)
	Rural area (<5000)	101 (19.0%)	81 (16.1%)	77 (14.7%)	180 (34.1%)	439 (21.0%)
Income	Below national average	163 (30.7%)	157 (31.2%)	83 (15.8%)	152 (28.8%)	555 (26.6%)
	Approx. average	205 (38.6%)	185 (36.7%)	220 (42.0%)	206 (39.0%)	816 (39.1%)
	Above national average	113 (21.3%)	100 (19.8%)	176 (33.6%)	105 (19.9%)	494 (23.7%)
	NA (no answer)	50 (9.4%)	62 (12.3%)	45 (8.6%)	65 (12.3%)	222 (10.6%)
Chronic disease	Yes	225 (42.4%)	273 (54.2%)	202 (38.5%)	203 (38.4%)	903 (43.3%)
	No	304 (57.3%)	231 (45.8%)	321 (61.3%)	325 (61.6%)	1181 (56.6%)

A majority (94.7%) of our responders use the internet on a daily basis, while only 4.4% on a weekly basis. Regarding previous online purchases of any product or service, 95.5% reported such experience (54.5% frequently, 32.4% several times, and 8.6% stating once or twice). Due to the online nature of our questionnaire, internet use and online shopping statistics are higher, yet comparable with recent EU statistics. According to Eurostat data (Eurostat, 2020), the frequency of daily internet use in Hungary is 82%, Czechia 81%, Slovakia 80%, and Poland 74%, and the proportion of individuals who ordered or purchased goods or services over the internet is 75% in Czechia and Slovakia, 66% in Hungary, and 61% in Poland. Our study focused on the impact regarding the COVID-19 pandemic, hence the time spent over the internet and attitude towards online purchases were measured using a five-point Likert scale with 1 indicating distinct “negative change,” 3 for “no change,” and 5 “positive change.” The same extent of positive change (Mean = 3.65, SD = 0.9) was observed for both values. In consideration of internet use, there was a country specific change during the COVID-19 pandemic. In all countries surveyed, individuals spent more time online, however, in both Hungary and Poland, this time frame increased more than in when compared with the Czech Republic and Slovakia [H (3) = 45.29 *p* < 0.05]. Notably, the positive attitude towards an online purchase has increased in all the four countries without any country specific patterns [H (3) = 7.25 *p* > 0.05].

### Attitudes toward buying medicines online

A vast majority (92.8%) of the respondents were aware medicines can be purchased online, with a significant difference between the examined countries [χ2 (3) = 12.13 *p* < 0.05]. Only 4.4% of respondents were unaware of this opportunity in Poland, while in Hungary, Czechia, and Slovakia these numbers were somewhat higher (6.3%, 9.0%, and 9.1%, respectively). Attitudes towards the three dominant supply channels of medicines have been assessed using a 5-point Likert scale, with 1 indicating “not appropriate at all” while 5 “totally appropriate”. Respondents considered community pharmacies as the most appropriate source when purchasing medications (Mean = 4.6 SD = 0.8), while they exhibited generally neutral attitudes towards non-pharmacy retail units, including petrol stations or herbal shops (Mean = 2.8 SD = 1.2), and the internet (Mean = 2.8 SD = 1.3). Despite minor differences in the evaluated countries, respondents find stationary pharmacies very appropriate with mean values above 4.5, while they reject the online channel with a mean value below 2.9 [H (3) = 4.29 *p* > 0.05]. However, attitudes towards using non-pharmacy units to purchase medications differ among the four countries surveyed. In both Hungary and Poland, individuals more often accepted these stores, when contrasted with individuals in Czech Republic and Slovakia who rated them lower [H (3) = 84.24 *p* < 0.05].

The questionnaire evaluated attitudes toward online medication purchases using a five-point Likert scale, in which a score of 1 was given for “I don’t agree” and 5 for “I agree”. In accordance with previously published results, convenience, 24/7 availability, and easy accessibility were mentioned as main benefits, meanwhile, inappropriate product information, abuse potential, and uncontrolled medication use were identified as the dominant risk factors. Two scales were developed based on the answers. The nine items of benefits comprised the Benefits scale (Cronbach’s *α* = 0.86) measuring how individuals rate the benefits of online medication purchases. The nine items of disadvantages comprised the Risks scale (Cronbach’s *α* = 0.9) measuring how individuals rate the risks of online medication purchases. The results show individuals in the Czech Republic and in Slovakia rate the benefits and risks on the same level without significant differences [CZ: t (530) = 0.12 *p* > 0.05; SK: t (527) = 1.57 *p* > 0.05]. In Hungary and in Poland, respondents rate the benefits higher than the risks [H: t (503) = 2.48 *p* < 0.05; PL: t (523) = 7.02 *p* < 0.05], showing a more positive attitude toward online medication purchases in these two countries. In evaluating the potential impact of perceived benefits and risks on a direct attitude toward online medications purchase (see Table 3.), the higher impact of benefits can be found on all three examined levels (pre-pandemic prior to May 2020, post-pandemic following 2020, and planned purchases following May 2022), however both benefits and risks show a significant correlation (*p* < 0.001).

**TABLE 3:** Results of the benefits and risks scales measuring how individuals rate online medication shopping on a five-point Likert scale (1 being “I don’t agree”, 5 “I agree”) and the Spearman’s rho correlation to the direct attitude toward online medications purchase.

Benefits	Statements for potential benefits	Mean value (SD)
Statements	Fast	3.56 (1.31)
	Convenient	4.18 (1.13)
	Inexpensive	3.18 (1.20)
	Products can be compared faster and more easily than in the pharmacy	3.46 (1.33)
	I can get more information compared to the pharmacy	3.05 (1.38)
	Individuals who can’t get to a pharmacy can also purchase products	4.11 (1.15)
	I can purchase medicines beyond opening hours	4.16 (1.16)
	I can access products which are otherwise not available to me	3.29 (1.36)
Sum of Benefits scale	Mean = 31.43, SD = 7.77, Min = 9 Max = 45	
Correlation of Benefits scale with direct attitudes toward online medication purchase		Correlation Coefficient (p)
Regularity prior to March 2020		0.371 (*p* < 0.001)
Regularity following March 2020		0.430 (*p* < 0.001)
Likelihood of purchasing in the future (in 2022)		0.552 (*p* < 0.001)
Risks	Statements for potential disadvantages	Mean value (SD)
Statements	I may not get the right product	3.33 (1.32)
	I do not get proper information regarding the use of the products	3.47 (1.31)
	The source of the product is not reliable or controlled	3.27 (1.31)
	It is easier to abuse preparations	3.66 (1.30)
	Due to the delivery time, I’m getting the drug later compared to a pharmacy	3.47 (1.26)
	There is no control, so I can get products I don’t need or worsens my condition	3.44 (1.32)
	It is hard for me to choose between the vast numbers of products	3.25 (1.28)
	The quality of the product is lower compared to those found in local pharmacies	2.71 (1.28)
	I may get illegal, substandard or falsified medicines	3.26 (1.38)
Sum of Risks Scale	Mean = 29.85.43 SD = 8.90 Min = 9 Max = 45	
Correlation of Risk scale with direct attitudes toward online medication purchase		Correlation Coefficient (p)
Regularity prior to March 2020		−0.315 (*p* < 0.001)
Regularity following March 2020		−0.370 (*p* < 0.001)
Likelihood of purchasing in the future (in 2022)		−0.474 (*p* < 0.001)

### Prevalence of use and the impact of COVID-19 on online purchases

**FIGURE 1:**
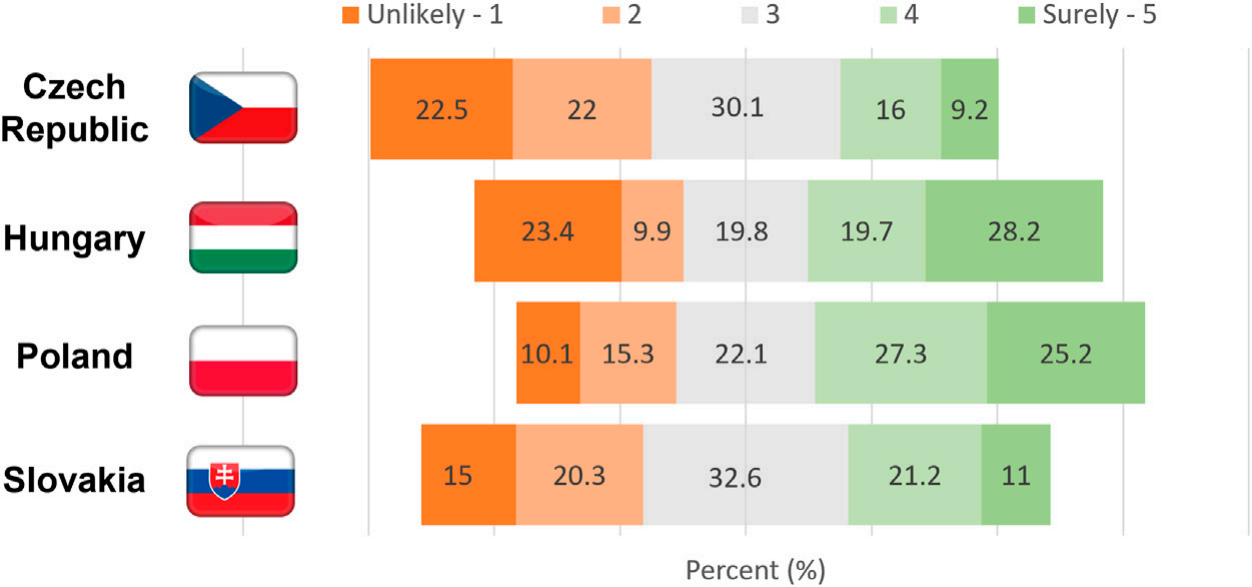
Likelihood of prospective medicine purchases over the internet in four central European countries.

An additional aim of the study was to evaluate the regularity of use and the impact of the pandemic regarding online medicine and health product (e.g., dietary-supplements and herbals) purchases. Prospective plans regarding how likely respondents purchased medicines over the internet following the time of our survey were evaluated using a five-point Likert scale (1 for “unlikely” and 5 for “surely”). The results depict the commitment towards purchasing medicines online, in general, and is very close to the neutral state (Mean = 3.05 SD = 1.35), yet there are significant differences among countries. In both the Czech Republic and in Slovakia, individuals slightly reject prospective online medication purchase, while respondents in Hungary and Poland slightly accept it [H (3) = 93.83 *p* < 0.05]. Thus, approximately one out of four Hungarian and Polish respondents were strongly committed towards purchasing medicines online, while contrasted with the Czech Republic and Slovakia in which only one out of ten will surely purchase online in the future. The frequency of unlikely (*n* = 370, 17.7%) and surely (*n* = 381, 18.3%) responses in the international dataset indicates approximately one out of five individuals will definitely purchase medications online in the evaluated central European countries, meanwhile, the same portion of individuals will not. Figure 1 illustrates differences of the country specific responses regarding the probability of prospective online medicine purchases.

**FIGURE 2:**
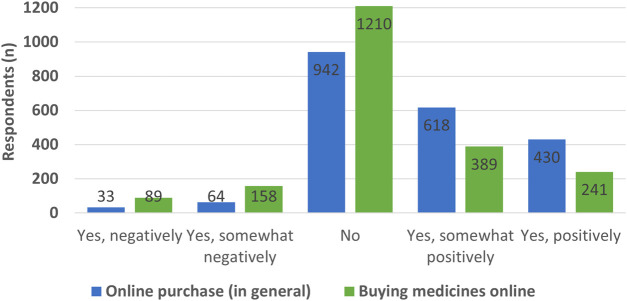
Opinion of respondents on the change of attitudes towards buying products (in general) or medicines online after the COVID-19 pandemic.

Distinctively, COVID-19 impacted consumers’ attitude towards internet pharmacies, aptly represented among 241 (11.5%) individuals of whom indicated their attitude change being positive and 389 (18.6%) somewhat positive. The majority (*n* = 1210, 58%) of the study population noted no attitude change, while the remainder, 11.9%, openly stated a negative attitude change (see Figure 2). Interestingly, both in the case of general goods and medications, the positive change in the attitudes is deemed significant [t (2086) = 33.044 *p* < 0.05; t (2086) = 12.86 *p* < 0.05, respectively]. Average attitude scores regarding the five-point Likert scale offers comparison of different variables. When the change in the populations’ attitudes since the outbreak of the COVID-19 pandemic is examined, it can be recognized individuals began investing considerable more time perusing the internet (Mean = 3.65 SD = 0.88) while, in parallel, their attitude toward purchasing both goods (Mean = 3.65 SD = 0.89) and medications (Mean = 3.26 SD = 0.91) online became increasingly positive. Admittedly, the positive change regarding medications is significantly smaller (Z = −16.84 *p* < 0.01). Even though there is a difference in the attitude levels toward purchasing goods and medications online in the four examined countries, our results demonstrate there is no significant difference in the extent of the tendency the positive attitude toward purchasing general goods increased more than the attitude toward purchasing medications online [F (3) = 1.13 *p* > 0.05].

**FIGURE 3:**
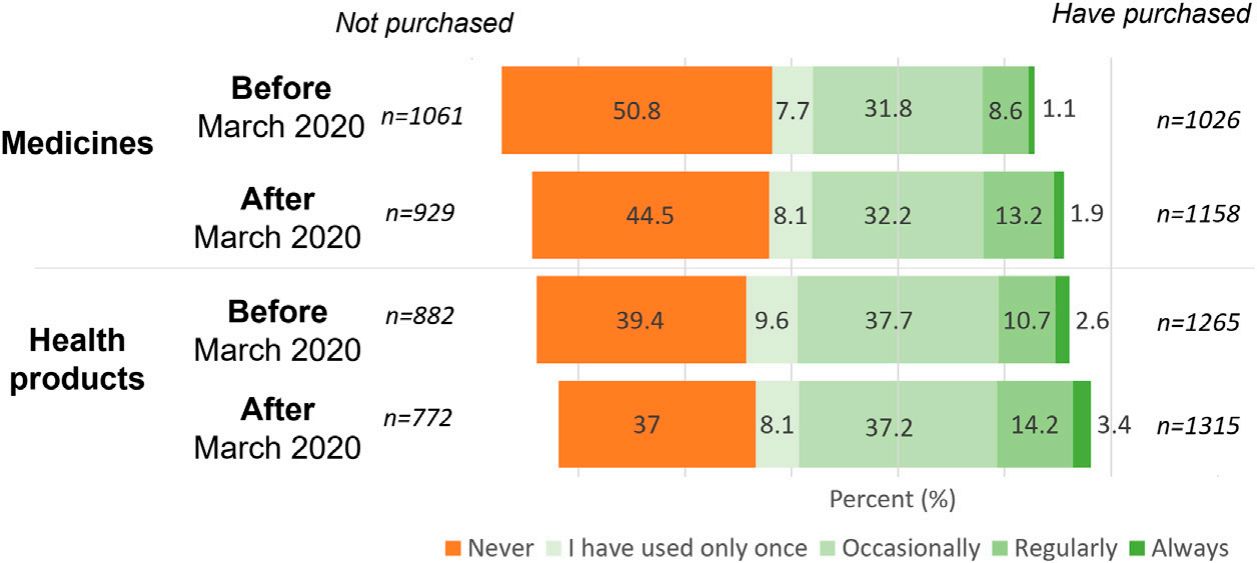
Frequency of medicine and health product (e.g. dietary supplements, herbal) purchases before and after March 2020 in Visegrad Group countries (*n* = 2087).

Regularity of previous purchases prior to and following the outbreak of the pandemic corresponds to the measured attitude changes. Participants were asked to indicate the frequency of purchasing medicines and health products (dietary supplements or herbals) prior to and following March 2020, using a five-point Likert scale (1 for “never” and 5 for “always”). There is a notable relative increase in the number of individuals purchasing medicines and health products online since the outbreak of the COVID-19 pandemic. Throughout the Visegrad Group countries, the number of individuals purchasing medicines online increased to 55.48%, which is a significant change compared to the pre-pandemic prevalence of 49.16% [χ2 (1) = 33.41 *p* < 0.05]. The case is very similar regarding health products with an increase from 60.66% to 63.01%, of which, is considered a significant change [χ2 (1) = 5.02 *p* < 0.05]. It is possible the level of change is smaller in this case due to the higher original ratio of online purchases of dietary supplements and herbals when compared to medications (60.61% vs. 49.16%). Additionally, a significant change appeared in both product categories regarding the frequency of online purchases. In both cases, the results demonstrated an increased frequency (medications: *Z* = −6.85 *p* < 0,05, health products: *Z* = −6.18 *p* < 0.05), see Figure 3. The changes in online purchases were analyzed on a country-specific level as seen in Table 4. Based on the results among the four countries, no difference appeared in the tendencies of purchase either in medicines [χ2 (3) = 0.21 *p* > 0.05] or health products [χ^2^ (3) = 0.11 *p* > 0.05].

**TABLE 4:** Prevalence of consumers purchasing medicines and health products over the internet prior to and following the outbreak of the COVD-19 pandemic.

Country	Online medicine buyers n (%)		Online health product buyers n (%)	
	Prior to March 2020	Since March 2020	Prior to March 2020	Since March 2020
Czech Republic	202 (38.04%)	251 (47.27%)	276 (51.98%)	294 (55.38%)
Hungary	223 (44.25%)	247 (49.01%)	306 (60.71%)	314 (62.30%)
Poland	300 (57.25%)	328 (62.6%)	324 (61.83%)	323 (61.64%)
Slovakia	301 (57.0%)	332 (62.88%)	359 (67.99%)	384 (72.72%)
Sum of V4 countries	1026 (49.16%)	1158 (55.48%)	1265 (60.61%)	1315 (63.01%)

## Discussion

In the recent past, online purchasing behavior has intensified due to external factors as the overall shift towards e-commerce was influenced by the pandemic and government restrictions (Gu et al., 2021). Evidently, the COVID-19 pandemic has significantly affected both the supply and demand side of the online pharmaceutical and healthcare product market, since the shopping experience among patients/consumers has simultaneously increased and their opinions regarding online purchase of medicines was likely impacted. Consumer perception regarding the online pharmaceutical market channel was evaluated from four perspectives in our study (i): awareness of and attitudes towards various channels of distribution (ii); complex attitude integrating nine potential benefits and risks (iii); frequency of medicine and health product purchases prior to and following the outbreak of the pandemic; and (iv), the likelihood of future online medicine purchases. A distinct increase in buying medicines and health products online was measured following the breakout of the COVID-19 pandemic, indicating one out of two respondents experienced online medicine purchases. Notably, 2 years since the outbreak and the end of external restrictions resulting in quarantine, individuals still prefer brick and mortar pharmacies.

Our research has shown most respondents (92.8%) are aware medicinal products can be purchased *via* the internet. However, 2 years following the global outbreak of the COVID-19 and following the fourth wave in Europe, most of these individuals still prefer to purchase medicines traditionally (offline) and consider community pharmacies as the most trustworthy source of medications. Interestingly, the proportion of individuals being aware of online pharmacies in our study was lowest in Czechia, despite market research showing e-commerce non-prescription market value share was the highest for this country in 2020 (IQVIA Consumer Health, 2021). Likely, conclusions on the utilization and attitudes regarding the online pharmacy channel should not be drawn by indirect economic analysis metrics (CAGR, market share, volume of sales), and more studies are necessary to understand how patients perceive this relatively new channel of distribution. According to our previous survey in Hungary, carried out in 2018, nearly 83% of participants indicated a high awareness regarding the possibility of ordering drugs online, yet only 4.17% of these individuals surveyed accessed this route to purchase medications in the past (Fittler et al., 2018). Behaviors of individuals who participated in the survey regarding consumer internet purchase of drugs in Malta in 2016 were similar, since 11.3% of respondents reported buying medical products online (Bowman et al., 2020). A recent study conducted in India showed more than 83% of respondents were aware of the online pharmacy, yet only slightly over 2% of the population surveyed prefer to purchase medicines through the internet (Bansal et al., 2022).

Our results indicate individuals began to allocate more time on the internet and their attitude toward purchasing both goods (in general) and medications online became more positive, however, this positive change regarding medications is significantly smaller. Seemingly, the COVID-19 pandemic has significantly increased the number of individuals purchasing medicines and health products online since the outbreak of the COVID-19 pandemic, indicating a tipping point as more than half of the participants in our survey experienced some level of online medicine purchasing. Reportedly, the impact of the pandemic remarkably affected online purchasing habits in other regions. The survey by Jairoun and colleagues (Jairoun et al., 2021), carried out in the United Arab Emirates, revealed 30% of respondents decided to purchase medications online due to the COVID-19 pandemic. Dietary supplements including analgesic, antihistamine, and cough medicines were primarily purchased by participants according to the study. In stark contrast, in consideration of published literature prior to the pandemic, only 1.4%–2.7% of the Saudi Arabia population purchased medication online in 2014 (Abanmy, 2017).

Notably, the prevalence of medicine purchased using the internet has increased from 4.17% in the Hungarian population in 2018 (Fittler et al., 2018) to 44.25% by March 2020, while 55.48% of survey participants stated they used the internet for medication purchases following the pandemic according to the findings of our current study. A report entitled, “COVID-19 and E-commerce” prepared by the United Nations Conference on Trade and Development, presented results of studies in which the effect of COVID-19 regarding the online purchasing behavior of consumers in China, Germany, Italy, Republic of Korea, Russian Federation, South Africa, Switzerland, Turkey, and separately, in Brazil, were examined. As noted, the e-sector representative of pharmacy and healthcare products is one of the categories, next to electronics, ICT (information and communications technology) products, gardening tools and “do-it-yourself,” with the largest growth of active users due to COVID-19 across all of these countries. The outcome of this report indicated the number of individuals who purchase medicinal products online at least once every 2 months, excluding Brazil, increased by 9% since the outbreak of COVID-19 (total sample *n* = 1819). In consideration of Brazilian consumers (*n* = 1878), the percentage was even greater, rising from 15% to 31% following the COVID-19 pandemic. Strikingly, the trend implies 1 in 3 individuals taking part in the survey in this country completed at least one purchase in the pharmacy and health e-sector since the COVID-19 outbreak (United Nations Conference on Trade and Development, 2020).

The major strength of our study is highlighted in being the first known publication providing evidence for utilization of internet pharmacy following the pandemic throughout Europe. Furthermore, no international study has been published referencing the prevalence of and attitudes towards purchasing medicines and health products *via* the online marketing channel. The representative sample of respondents throughout four central European countries permits the extrapolation of our findings to a larger population in the Visegrad Group countries. Admittedly, our study bears several limitations. Although the majority of the central European population use the internet regularly, our findings do not represent the smaller portion of individuals who do not use the internet. Uncontrolled sampling and survey bias must be noted due to the online nature of our survey. Although the majority of medicine consumers are seniors, internet users tend to be younger. This survey bias is reflected in the current sample with a mean age of 38.3 years. Additionally, since demographic characteristics of the countries are not identical, cross-country comparison may be biased to a limited extent. As the legislative background of the observed Visegrad Group countries permits only non-prescription medicines to be sold *via* the internet, we can assume most respondents have purchased non-prescription products. Consequently, increased utilization of online pharmacies for prescription medicines cannot be directly concluded from our findings.

Internet pharmacies vary in levels of quality. The global internet pharmacy marketplace, due to lack of international synchronized regulations and effective law enforcement, bears numerous patient and medication safety issues. According to the literature data about 10% of medicines worldwide could be substandard or falsified (SF) (Ahmed et al., 2022). The problem may be related to both an active substance (its quality and dose) and excipients. Consequently, such drugs may not produce the desired pharmacological effect. Although most of the SF medicinal products are identified in low- and middle-income countries, the internet seems to be a perfect environment to distribute them. Cybercriminals utilize online platforms since they have an easy access to customers when at the same time, they stay relatively anonymous. However, it should be emphasized, legitimate online retailers of medicines throughout Hungary, Poland, Czechia and Slovakia are regulated in the same way in the sale of medicines at community pharmacies. Only registered brick and mortar community pharmacies can legally offer medicines online and medicines which require a physician’s prescription can be sold only at stationary pharmacies according to regulations applicable in the Visegrad Group countries. Thus, medicines purchased are relatively safe, although an inappropriate self-diagnosis assumed by the patient or, the lack of patient’s awareness related to drug interactions or drug contraindications can impact patient safety (Mazer et al., 2012; Fittler et al., 2018; Oleszkiewicz et al., 2021). When browsing the internet, one can find plenty of illegal sites (often based abroad to avoid local jurisdiction) offering prescription-only medicines without a valid medical prescription. Patients may be attracted by bargains, uncontrolled access and anonymity when purchasing medicines from unapproved online medicine retailers. Although illegal internet pharmacy websites are considered the dominant source of substandard and falsified medications among developed countries (Mackey and Nayyar, 2016), limited and controversial real-world data is available regarding the prevalence of such dubious products and their direct health consequences (Pozsgai et al., 2022). In order to ensure adequate safety when purchasing medicinal products online, drug authorities including the European Medicines Agency and the US Food and Drug Administration provide guidelines referencing the most important principles which should be followed by patients when making such purchases to increase consumer awareness (US Food and Drug Administration, 2018; European Medicines Agency, 2022). Moreover, the European Commission introduced a common logo that allows patients to identify authorized online pharmacies selling authentic medicines.

At the tipping point towards the new normal, as the prevalence of e-pharmacy shopping constantly increases, there is a need in disseminating public information campaigns addressing internet users to drive their awareness to risks related to purchasing medicines online from unknown sources and prevent the use of unregistered, substandard and falsified medicinal products. Public awareness campaigns are needed to persuade internet shoppers to evaluate drug selling websites, avoiding ones with low credibility and preferring nationally approved verified retailers.

## Data Availability

Survey instrument in English and data supporting the findings of this study are available from Mendeley Data at https://data.mendeley.com/datasets/jf6k3vmrg6 or from the corresponding author AF on request.
